# An application of RASER technique in the treatment of chronic total occlusion accompanied with stent fracture in right coronary artery: a case report

**DOI:** 10.1186/s12872-019-1258-1

**Published:** 2019-11-29

**Authors:** Yajun Xue, Boda Zhou, Weimin Wang, Guobin Miao, Ou Zhang, Jie Zhou, Yu Geng, Yanlong Zhai, Chunhui Ren, Ping Zhang

**Affiliations:** 1 Department of Cardiology, Beijing Tsinghua Changgung Hospital, Beijing, 102218 China; 2 Department of Cardiology, Peking University People’s Hospital, Beijing, 010010 China

**Keywords:** Excimer laser coronary atherectomy, Rotational atherectomy, Chronic total occlusions, RASER technique

## Abstract

**Background:**

The interventional treatment of chronic total occlusion (CTO) with stent fracture as well as severe calcification was extremely difficult and no effective technique has been reported.

**Case presentation:**

A 50-year-old woman was hospitalized for angina, angiography revealed triple vessel disease, CTO accompanied with stent fracture in right coronary artery (RCA). Treatment using conventional coronary intervention was expected to be difficult. Therefore, we performed RASER technique, which was a combination of excimer laser coronary atherectomy (ELCA) with rotational atherectomy (RA), followed by the deployment of drug-eluting stents. Intravascular ultrasound (IVUS) revealed well attachment of the stents, the patient was discharged 3 days after the procedure and no recurrent chest discomfort was reported in a follow-up time of 10 months.

**Conclusion:**

This case report provided a first report of RASER technique in the treatment of CTO with stent fracture and severe calcification.

## Background

Although drug-eluting stent based intervention has become the most widely used treatment for coronary heart disease, a high incidence of in-stent restenosis after coronary stent implantation remains an important challenge [1]. Previous studies demonstrated that rotational atherectomy (RA) is safe and effective to treat stent restenosis [2, 3]. Meanwhile, excimer laser coronary atherectomy (ELCA) has a long history of adjunctive therapy that can be applied to treat in-stent restenosis (ISR) [4]. However, in particular case of complicated CTO after ISR, microcatheter and Rota Wire could not pass the lesion, makes the operation difficult. The RASER technique combines ELCA with RA, since ELCA could provide an upstream channel to permit microcatheter and Rota Wire passage, while RA could fully debulk the lesion [5]. Unfortunately, there is no report regarding combination of ELCA and RA in CTO accompanied with stent fracture. In the current report, we described a novel application of RASER technique to successfully treat in-stent occlusion accompanied with stent fracture in RCA.

## Case report

**Fig. 1:**
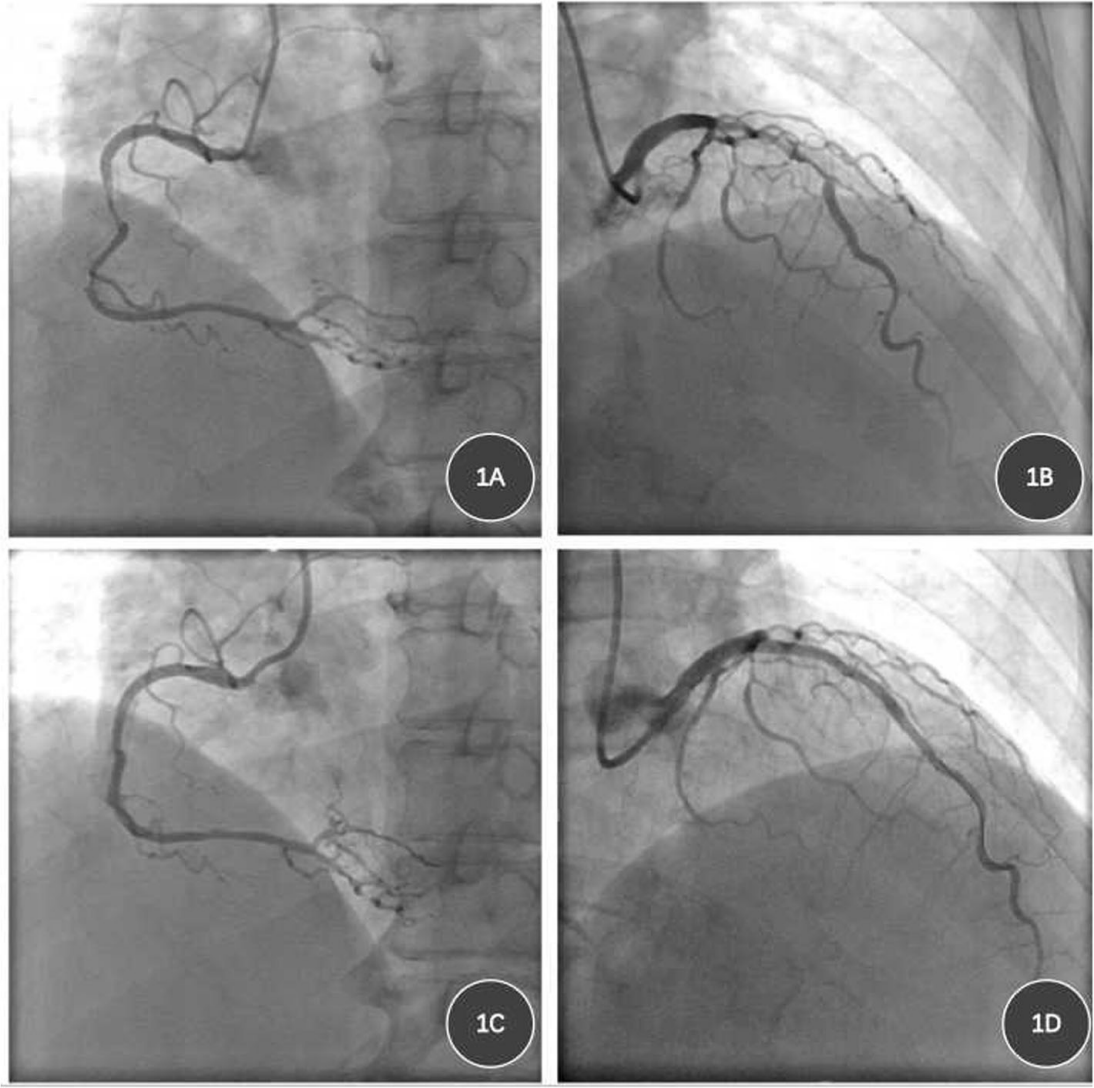
Images of coronary angiography and PCI 4 years ago. **a**, severe and diffused stenosis in the middle segment of RCA; **b**, severe and diffused stenosis in the proximal segment of LAD; **c**, postoperative angiography of RCA; **d**, postoperative angiography of LAD

A 50-year-old woman was hospitalized on October, 2017 for ongoing limiting angina pectoris. The patient had a history of precordial chest pain for 4 years, and aggravation for 1 week. Four years ago, she was diagnosed unstable angina pectoris and old anterior myocardial infarction at local hospital. Angiography revealed triple vessel disease involving proximal LAD, middle LCX and middle RCA (Fig. 1), The patient underwent percutaneous coronary intervention (PCI) with 3 drug eluting stents (DESs) (2.5 mm × 33 mm, 2.5 mm × 18 mm and 2.5 mm × 23 mm) in RCA, and 1 DES (3 mm × 38 mm) in the LAD (Fig. 1). The patient’s symptoms relived after PCI, while secondary prevention medications were taken regularly (clopidogrel was stopped after one and a half years, aspirin continued). Past medical history includes type 2 diabetes for 21 years, hyperlipidemia for 4 years, left renal artery stenosis and underwent stenting for 1 year, diabetic foot necrosis and underwent left foot amputation for 5 months. She has no family history of coronary heart disease.

**Fig. 2:**
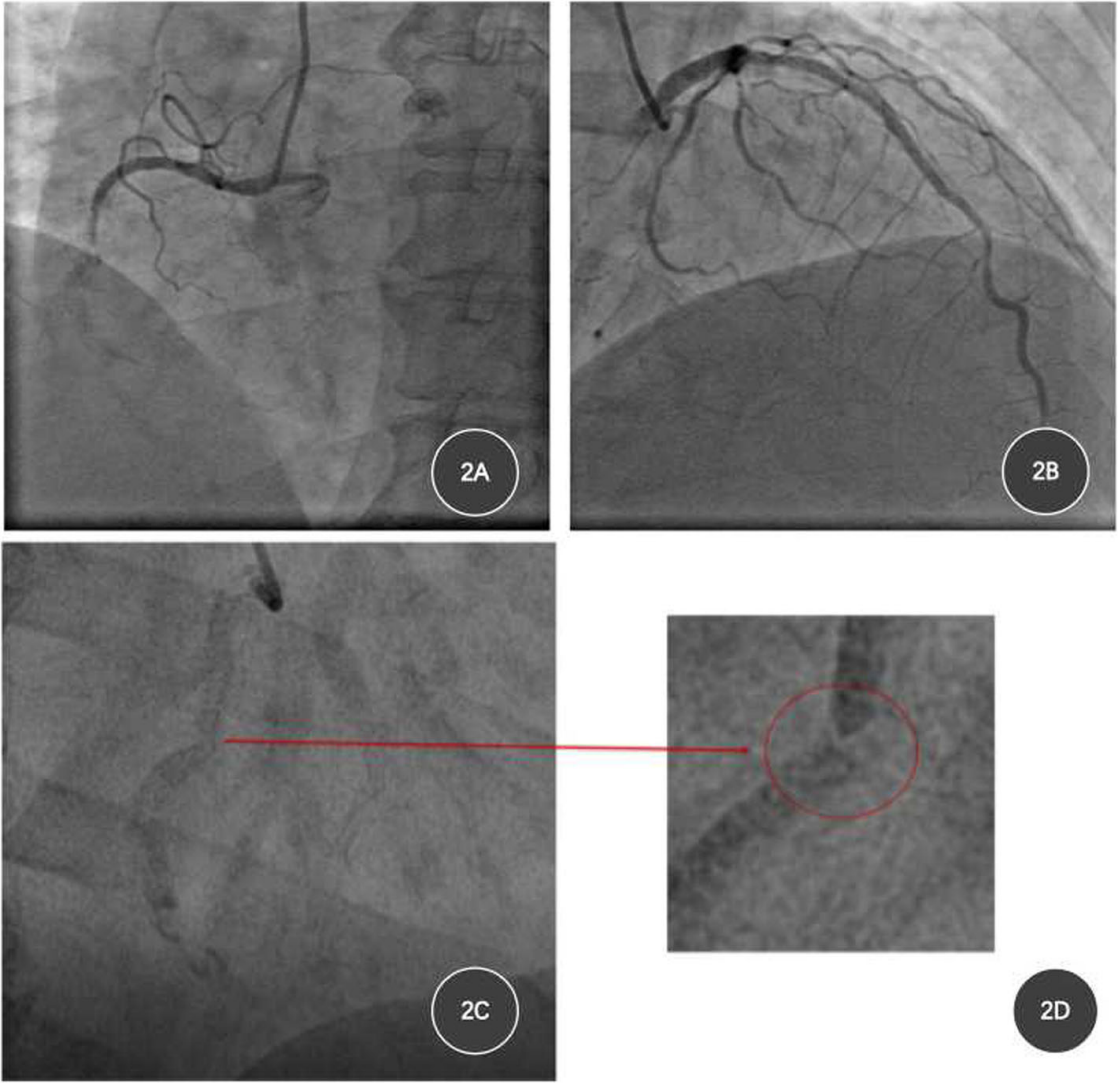
Images of coronary angiography after hospitalization. **a**, stents in proximal and middle RCA, total occlusion in middle RCA; **b**, stent in proximal LAD, accompanied with mild intimal hyperplasia in stent, 50–70% diffused stenosis in middle LCX and 70–85% diffused stenosis in proximal and middle OM2; **c** and **d**, stent fracture at the second turning point of RCA

After hospitalization, physical examination was non-remarkable, and secondary prevention treatment was prescribed, troponin was negative, echocardiography revealed LVEF 45%. Coronary angiography revealed visible stents in the proximal and middle segments of RCA, with in-stent total occlusion (Fig. 2a), stent fracture could be seen at the second turning point of RCA (Fig. 2c and d). Stent was seen in proximal LAD, with mild intimal hyperplasia in the stent (50–60% diffused stenosis), 50–70% diffused stenosis in middle LCX and 70–85% diffused stenosis in proximal and middle of second obtuse marginal artery (OM2) (Fig. 2b). The patient was diagnosed unstable angina pectoris and old anterior myocardial infarction, and PCI was indicated.

**Fig. 3:**
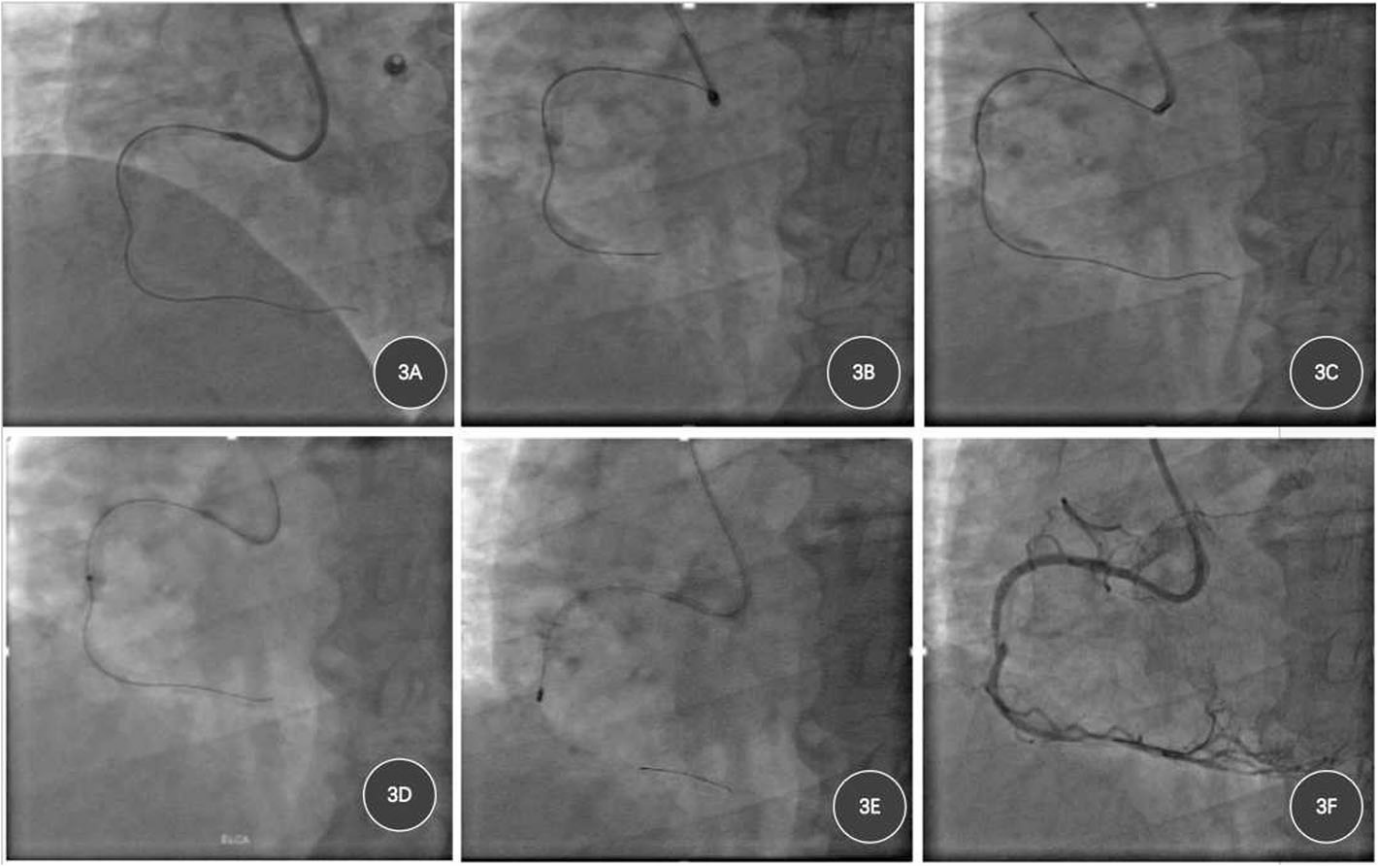
Coronary intervention process in RCA. **a**, GAIA second guidewire traversed the CTO lesion in RCA; **b**, Finecross microcatheter could not pass the occlusion, nor could Corsair microcatheter; **c**, application of balloon anchored guidewire technique still failed to pass the occlusion; **d**, ELCA was performed using a 1.4-mm burr excimer laser catheter to ablate in-stent occlusion; **e**, RA was performed using a 1.25-mm burr RotaLink at a speed of 170,000 r/min; **f**, coronary angiography after ELCA and rotational ablation

**Fig. 4:**
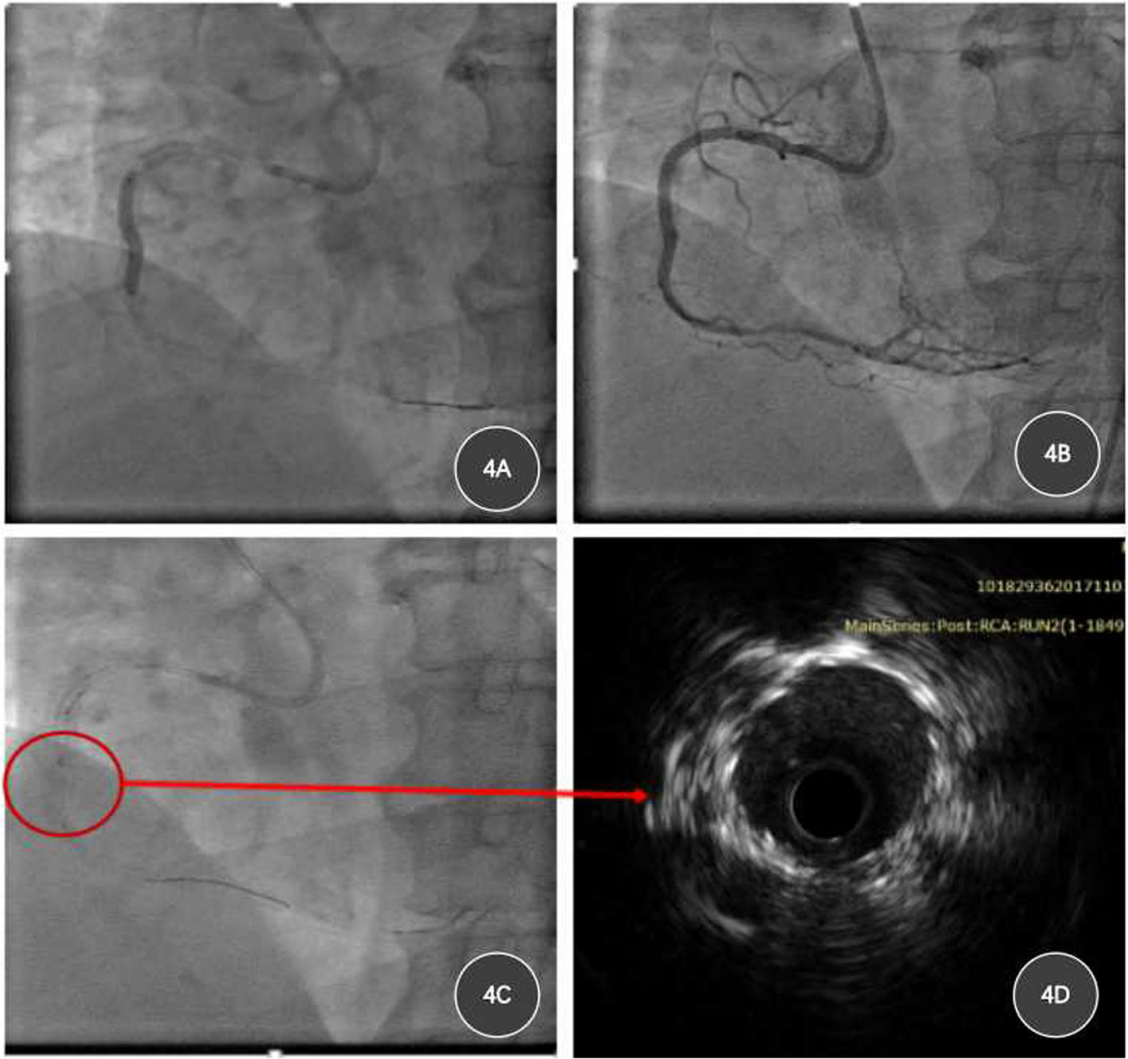
Stent implantation and IVUS examination of RCA. **a**, a 2.75 mm*24 mm DES was deployed in RCA; **b**, coronary angiography after the deployment of stent; **c** and **d** IVUS revealed well attachment of stent to the vessel wall

As the ISR of RCA was CTO, which accompanied stent fracture, two difficulties present: ①it will be hard for the guidewire to traverse the lesion through true lumen. ②balloon expansion in the stent fracture region was expected to be difficult. Therefore, we performed transradial PCI using 6F AL1.0 guiding catheter, after GAIA second guidewire (Asahi Intec, Abbott Vascular, Rangendingen, Germany) traversed the lesion, neither microcatheter nor balloon anchored guidewire could pass the leision, so we used a 1.4 mm ELCA catheter (CVX-300, Spectranetics, CO, USA) with a pulse rate of 40 Hz and energy output of 45 mJ/mm^2^ to ablate ISR for 3 times (Fig. 3d). Then a Finecross MG catheter (Terumo Medical Corp., NJ, USA; 1.8 Fr) was applied to exchange for the Rota Wire, so that Rota Wire could be easily advanced to distal RCA. Then, RA was performed using a 1.25-mm burr (RotaLink, Boston Scientific) at the speed of 170,000 r/min for 75 s to fully debulk the lesion (Fig. 3e and f), IVUS showed 90°-270° calcification and stent fracture (Additional file 1: Figure S1), followed by the deployment of one drug-eluting stent (2.75 mm*24 mm, Fig. 4a and b). Intravascular ultrasound (IVUS) revealed well attachment of the stent (Fig. 4c and d), and the patient was discharged without complication 3 days later, echocardiography revealed LVEF 60%(Additional file 2: Figure S2). The patient was followed up for 10 months and no recurrent chest discomfort was reported, no adverse event was reported, echocardiography revealed LVEF 64%.

## Discussion and conclusion

In this case, we successfully performed RASER technique using ELCA for ISR ablation combined with RA for rotational ablation in a patient with CTO in RCA accompanied with stent fracture. The RASER technique achieved excellent angiographic and IVUS result, the patient recovered well after the procedure and was discharged 3 days later.

ELCA is a long-established adjunctive therapy that can be applied during complicated PCI. The indications of ELCA during PCI include thrombus, non-crossable or non-expandable lesions, chronic total occlusions, in-stent restenosis and stent under-expansion [6]. The key advantage of ELCA over alternative atherectomy interventions is delivery on a standard 0.014-in. guidewire. The major limitation of ELCA is incompetency to ablate heavy calcification. In our case, CTO in RCA accompanied with stent fracture. Microcatheter could not pass the occlusion, indicating fibro-calcific nature of the lesion, which makes balloon dialation and rota ablation impossible. ELCA successfully created a channel after guidewire traversed the CTO lesion, which facilitated the passage of microcatheter and subsequent exchange of Rota Wire with guidewire. As the lesion was severely calcific, rotational atherectomy was necessary to fully debulk the lesion.

It was reported that longitudinal stent ablation by RA with a 1.75-mm burr was effective in treating under-expanded stent in CTO lesion with severe calcification [7]. However, inability of Rota Wire to pass the lesion was a major problem. In addition, ELCA has been recognized as an alternative to treat ISR [8], but severe calcific lesion frequently led to unsatisfactory result treating with ELCA. Therefore, RASER technique, a combination of ELCA and RA was the optimal choice for CTO lesion with severe calcification. However, the application of RASER in CTO with stent fracture as well as severe calcification has not been reported before, our case received a satisfactory result.

The ELCA catheters can be delivered with a standard 0.014″ guidewire and 0.9 mm, 1.4 mm, 1.7 mm, and 2.0 mm catheters are available. Studies showed that 0.9 and 1.4 mm catheters are more frequently used in CTO cases [9]. In the present case, we performed a 1.4-mm burr excimer laser catheter with a pulse rate of 40 Hz and energy output of 45 mJ/mm^2^ to ablate occlusion for 3 times. Alternatively, a 0.9-mm ELCA catheter is suggested when the lesion is severely occluded accompanied with calcification [10].

In summary, this is the first report of successful application of RASER technology in patients with CTO accompanied with severe calcification and stent fracture. This case report may extend ELCA indications and provide a novel approach to complicated PCI. Eventually, with the increase of cases and the accumulation of experience, patients who were unable to undergo conventional PCI will benefit.

## Supplementary information

**Additional file 1: Figure S1.** IVUS examination of RCA after RASER technique.

**Additional file 2: Figure S2.** Representative images of echocardiography before and 3 days after intervention.

## Data Availability

Data sharing is not applicable to this article as no datasets were generated or analysed during the current study.

## Abbreviations

CTO: Chronic total occlusion
DES: Drug eluting stent
ELCA: Excimer laser coronary atherectomy
ISR: In-stent restenosis
IVUS: Intravascular ultrasound
OM2: Second obtuse marginal
PCI: Percutaneous coronary intervention
RA: Rotational atherectomy
RCA: Right coronary artery
