# Supplementary figures and images for: An application of RASER technique in the treatment of chronic total occlusion accompanied with stent fracture in right coronary artery: a case report
Source: BMC Cardiovasc Disord. 2019 Nov 29;19:273. doi: 10.1186/s12872-019-1258-1 (PMC6883533; doi:10.1186/s12872-019-1258-1)

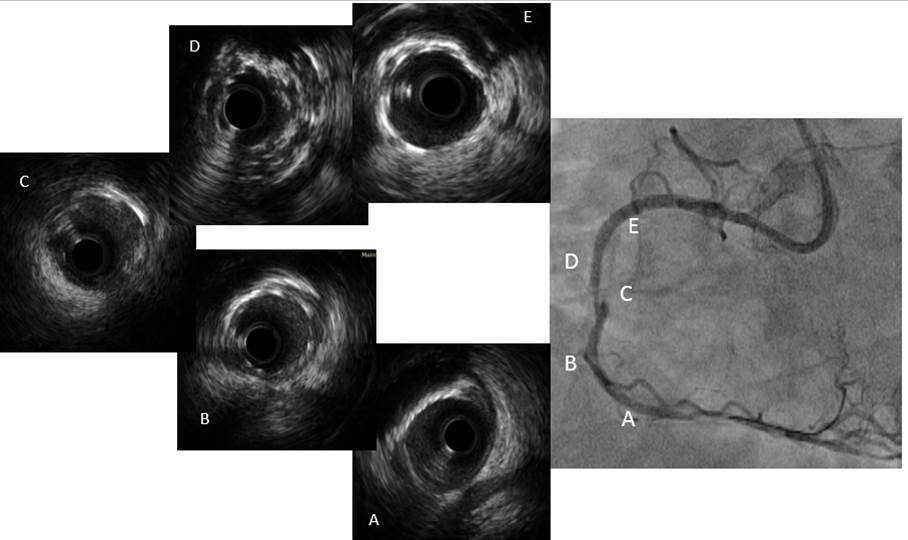

Supplement: Supplementary file 1 — Additional file 1: Figure S1. IVUS examination of RCA after RASER technique. [file 12872_2019_1258_MOESM1_ESM.jpg]

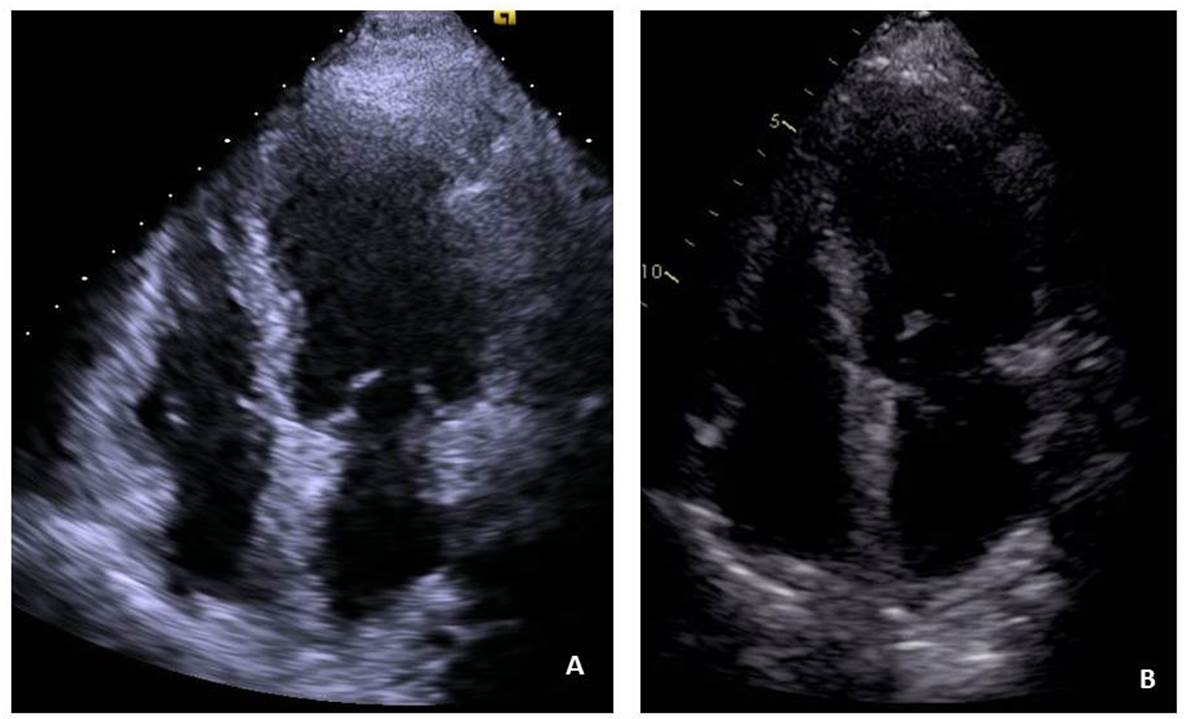

Supplement: Supplementary file 2 — Additional file 2: Figure S2. Representative images of echocardiography before and 3 days after intervention. [file 12872_2019_1258_MOESM2_ESM.jpg]
